# Investigation of Using Calcined Coal Gangue as the Co-Blended Precursor in the Alkali-Activated Metakaolin

**DOI:** 10.3390/ma17143610

**Published:** 2024-07-22

**Authors:** Ye Pan, Zichen Lu, Liheng Zhang, Hui Zhang, Qin Zhang, Zhenping Sun

**Affiliations:** 1 Key Laboratory of Advanced Civil Engineering Materials of Ministry of Education, School of Materials Science and Engineering, Tongji University, Shanghai 201804, China; 2130616@tongji.edu.cn (Y.P.); 2210579@tongji.edu.cn (L.Z.); szhp@tongji.edu.cn (Z.S.); 2 Shanxi Jiawei New Material Co., Ltd., Yuncheng 044200, China; jiawei@jiaweigf.com (H.Z.); ztsjsgssyzx@163.com (Q.Z.)

**Keywords:** calcined coal gangue, metakaolin, alkali-activated materials, flowability, mechanical properties

## Abstract

The feasibility and performance of using calcined coal gangue (CCG) to substitute metakaolin (MK) as the precursor to prepare alkali-activated materials (AAMs) were thoroughly evaluated by conducting combined experiments of flowability test, mechanical measurement, calorimetry and microstructure analysis, etc. It was found that the increased substitution ratio of CCG to MK can increase the flowability of the prepared paste by up to 28.1% and decrease its viscosity by up to 55.8%. In addition, a prolonged setting time of up to 31.8% was found with the increased substitution amount of CCG to MK, which can be attributed to the low reactivity of CCG compared to that of MK. Lastly, even though the presence of CCG can lead to a decrease in the early compressive strength of the hardened paste, a highly recovered long-term mechanical property can be found due to the continuous reaction of CCG. All of these results prove the feasibility of using CCG as one co-blended precursor with MK to prepare alkali-activated materials.

## 1. Introduction

As the largest industrial waste in China, coal gangue (CG) has a reserve of more than 6 billion tons [1,2,3,4]. Considering its huge damage to the environment caused by the massive waste dump, it becomes urgent to efficiently utilize this solid waste [5,6,7,8]. However, the reactivity of CG is quite low [9]. Hence, many methods have been adopted to improve the activity of gangue, such as mechanical grinding, chemical modification, thermal activation, microwave radiation, etc. [9,10]. Among these methods, thermal activation is the most commonly used and effective way to improve the reactivity of CG. Many studies have found that the pozzolanic activity of CG can be greatly improved after calcination at 700 °C for 2 h [11,12]. In addition, it was reported that the replacement of clinker by calcined coal gangue (CCG) can reduce the value of the global warming potential from 855 kg CO_2_-eq to 796.2 kg CO_2_-eq [13].

Considering the fast development of alkali-activated materials (AAMs) as low-carbon cementitious materials [14,15], one commonly accepted way to consume the massive amount of CG is to use it as precursor in AAMs after calcination. Many researchers have investigated the possibility of using CCG in AAMs. Cheng et al. [11] explored the effect of sodium hydroxide modulus and alkali lye amount on the strength and microstructure of alkali-activated CCG; the results show that the optimal sodium silicate modulus is 1.5~2, and the suitable NaOH concentration could be 12 mol/L or 14 mol/L. Wang et al. [1] studied the influence of CCG on the properties of alkali-activated fly ash by replacing 30% fly ash and concluded that CCG has a higher pozzolanic activity compared to fly ash and can be used for the substitution for fly ash without compromising the compressive strength of AAMs. Ma et al. [16,17] studied the effect of shrinkage-reducing admixture (SRA) on drying shrinkage and microstructural development of alkali-activated coal gangue and slag and found that the increased CCG content benefits the anti-chloride penetration of AAMs. Zhang et al. [18] investigated the effect of CCG on the mechanical properties, frost resistance, and chloride penetration resistance of alkali-activated slag; the results show that the sample with 50% CCG has the optimal property. Hence, CCG could be a possible precursor with a promising application used in alkali-activated materials.

Even though many investigations have been conducted on the combined effect of CCG with other precursors, such as fly ash and slag, it should be noted that the reserves for fly ash and slag are decreased due to the limited production amount of steel and the decreased consumption amount of coal [19,20]. In contrast, metakaolin (MK), as a widely used precursor of AAMs with high reactivity [14], has remarkably rich reserves in China [21]. By reacting with alkali, the activated metakaolin can lead to the formation of reaction products with outstanding properties such as high mechanical properties [22], superior resistance to chemical attacks [23], chloride ion penetration resistance [24], and frost resistance [25]. However, MK has some disadvantages, such as a high cost, high water demand, etc., which limit the wide application of metakaolin [26,27,28]. Additionally, compared to CG, kaolinite, as the raw material to prepare MK, generally has an important usage with high economic values; for example, in the sintering of ceramics. Hence, it could be a win–win strategy to combine the high activity of MK and the economic and environmental benefits of CCG. However, from the author’s knowledge, quite limited research was conducted to investigate the combined performance of the alkali-activated CCG and MK.

In this study, the calcined gangue was used to partially replace MK, and the performance of alkali-activated metakaolin blended with different amounts of CCG was investigated, including the rheological behavior, setting time, and compressive strength. Furthermore, calorimetry, X-ray diffraction (XRD), thermogravimetric analysis (TGA), and a mercury intrusion porosimetry (MIP) test were applied to better understand its reaction process and the development of microstructure. The results shown in this study not only provide a detailed evaluation of the performance of this new precursor used in AAMs but also guide the resource utilization of CG.

## 2. Materials and Methods

### 2.1. Materials

**Figure 1:**
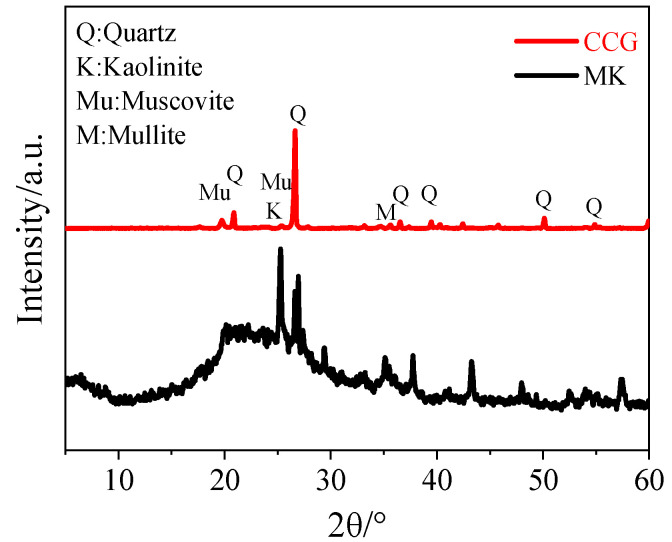
XRD patterns of MK and CCG.

Metakaolin (MK) and CG powder were purchased from Qiangdong Mineral Products Processing Factory, Shanxi, China. CCG was obtained by calcinating CG powder at 700 °C for 2 h in a muffle furnace. The chemical compositions of MK and CCG detected by X-ray fluorescence (XRF) are given in Table 1. The loss of ignition (LOI) was measured by using thermogravimetric analysis (TGA, Netzsch STA 409, Selb, Germany). The samples were continuously heated at 950 °C for 20 min, and the loss of mass was recorded as the LOI. The X-ray diffraction (XRD) pattern of raw materials in Figure 1 was characterized at a scanning rate of 2°/min from 5° to 60° 2θ. A broad hump ranging from 15° to 30° 2θ in Figure 1 indicates the amorphous state of metakaolin. In addition, the existence of quartz and muscovite can be detected in CCG [29]. The particle size of the MK and CCG was detected by a laser particle sizer (Mastersizer 3000, Malvern, UK), and the results are shown in Table 2. Alkali activator (modulus = 1.5, pH = 13.42) was prepared by blending 84.10 g sodium silicate solution (modulus = 3.3, solid content = 34%) with 10.56 g sodium hydroxide and 4.5 g DI water; the final sodium silicate solution (23.4% SiO_2_, 16.1% Na_2_O, 60.5% H_2_O) was prepared and used in this study.

### 2.2. Sample Preparation

As shown in Table 3, an increased substitution ratio of CCG from 0% to 50% (by mass) was used to prepare the AAM samples. The Na_2_O/binder ratio and water/binder ratio were selected as 0.15 and 0.6 (by mass), respectively. All the fresh AAM pastes were prepared according to [30]. Afterwards, the fresh AAM pastes were poured into plastic molds with a dimension of 40 mm × 40 mm × 40 mm and cured at the standard curing condition (the temperature and humidity in the standard curing room are 20 °C and 95% RH, respectively) for 3, 7, and 28 days. Then, the samples were crushed into small pieces and immersed in isopropanol for 2 d to stop the reaction. At last, the samples were dried in a vacuum oven at 40 °C and then ground into powder with a size of <75 μm for further experiments. 

### 2.3. Testing Methods

#### 2.3.1. Performance Evaluation

**Figure 2:**
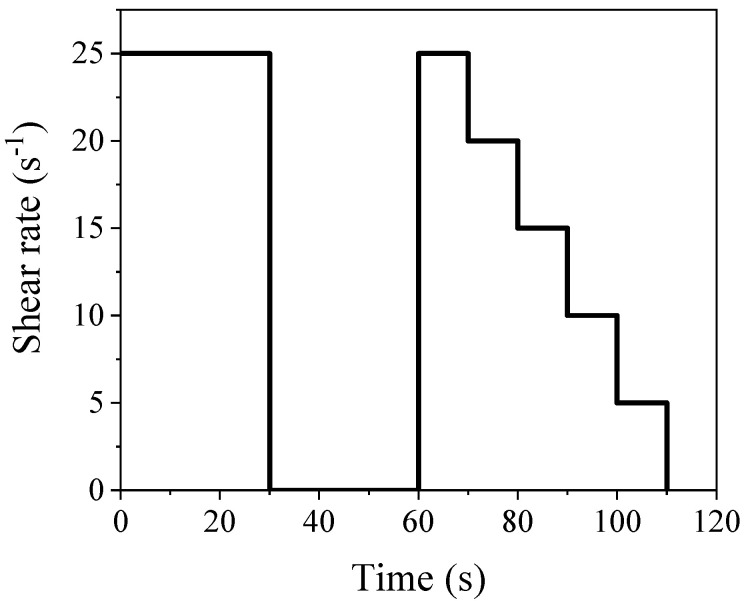
Test procedure of rheological test.

The flowability and its retention ability were studied by a flow table test and rheological measurement. The flow table tests for the samples after curing for 5 min, 1 h, and 2 h were conducted according to [31]. The rheological measurement was performed by using a rheometer with the Couette geometry (TR-MRI, Tongrui Instrument Company, Shanghai, China). The specific procedure for the measurement is shown in Figure 2. The Herschel–Buckley model (H-B model, Equation (1)) was used to obtain the rheological parameters, where τ, $\tau_{0}$, k, $\dot{\gamma }$, and n are the shear stress, yield stress, consistency indices, shear rate, and rheological indices, respectively [32,33].

(1) $$\tau =\tau_{0}+k{\dot{\gamma }}^{n}$$

The initial and final setting times were tested according to [30]. The compressive strength of the hardened pastes was measured after curing for 3, 7, and 28 d by an automatic compression test machine (YAW-300c, Yixuan Testing Instrument Co., Ltd, Cangzhou, China). Six samples were measured at each age, and the average value was used to represent the strength. 

#### 2.3.2. Reaction and Reaction Products

The reaction process was evaluated by using the TAM-Air isothermal calorimeter (TA instrument, New Castle, DE, USA) at 25 °C for 3 days. In total, 5 g of binder (namely MK and CCG) and the corresponding amount of activator and DI water were stored at 25 °C for one day to ensure the same temperature of the materials and environment. Then, the water and activator were added into an ampoule with the binder inside and stirred for 2 min by using a vortex mixer (MX-S, DLAB, Shanghai, China) to reach the homogeneity of the pastes. Afterwards, the ampoule was inserted into the device and the measurement was started to record the heat flow. 

XRD analysis was performed via an X-ray diffractometer (RIGAKU, model D/max, Tokyo, Japan) for samples after curing for 3 d and 28 d. The scanning range is from 5° to 60° and the scanning speed is 2°/min. In addition, the reaction products at the same curing age were analyzed by TGA. Samples of 10 mg were heated at a constant heating rate of 10 °C/min from room temperature to 900 °C. 

#### 2.3.3. Microstructure Analysis

The hardened AAMs were fractured into small blocks with a diameter of 3–5 mm and thickness of 1–2 mm and were used for the mercury intrusion porosimetry (AutoPore IV 9510, Micromeritics, Norcross, GA, USA) after curing for 3 d and 28 d. The maximum pressure p_max_ is 200 MPa. The pore diameter distribution curves of the samples were recorded by changing the mercury injection pressure. Additionally, the microstructure of the samples after curing for 3 d and 28 d was observed by using a Scanning Electron Microscope (SEM, ZEISS Gemin 300, Oberkochen, Germany). 

## 3. Results and Discussion

### 3.1. Flowability and Rheological Properties

**Figure 3:**
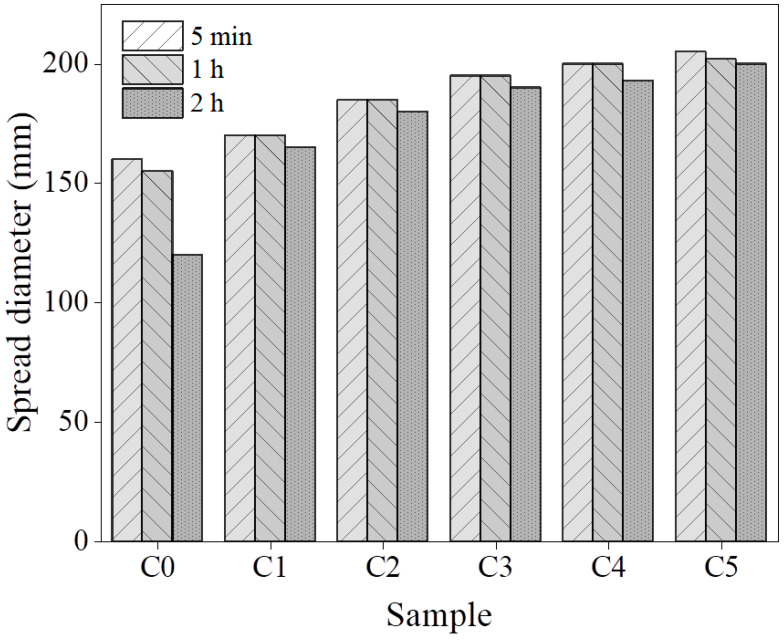
Flowability of the pastes after preparing for 5 min, 1 h, and 2 h.

Figure 3 presents the influence of different CCG substitution ratios on the flowability of the pastes after the preparation of 5 min, 1 h, and 2 h. With the increase in CCG’s substitution ratio, the flowability of the paste at 5 min increased from the initial 160 mm to 205 mm (28.1%) step by step, which indicates that the co-blending of CCG with MK is beneficial to increase flowability. Regarding the ability of fluidity retention, an obvious decrease in the spread diameter of C0 can be observed after curing for 2 h, which could be caused by the reaction of MK under the activation of alkali. However, it is interesting to notice that along with the increased substitution ratio of CCG, an obvious reduction in the loss of diameter was found, which indicates that the use of CCG is beneficial to increase the retention ability of AAMs due to their low pozzolanic activity compared to MK [34].

**Figure 4:**
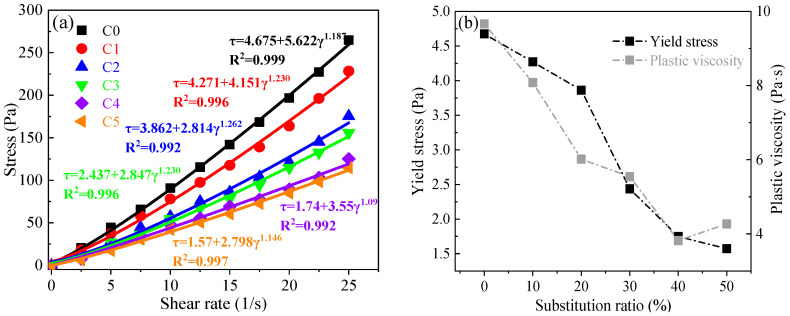
(**a**) Shear rate–shear stress curves of pastes prepared for 5 min; (**b**) the calculated yield stress and viscosity.

Figure 4 depicts the shear stress curves of the pastes after preparing for 5 min with different substitution ratios of CCG. In addition, the rheological parameters were calculated based on the fitting of the curves by applying the H-B model. As shown in Figure 4a, a decreased slope was found with the increased amount of CCG in the AAMs, which indicates that the viscosity of the prepared paste decreased with the increased amount of CCG. In addition, the power law index n is always higher than one for all the pastes, which indicates that the pastes are shear-thickening fluids [33]. The calculated viscosity and the yield stress are shown in Figure 4b. It can be found that along with the increased amount of CCG, the yield stress and plastic viscosity of the pastes were decreased from 4.68 Pa and 9.66 Pa·s to 1.57 Pa and 4.27 Pa·s (55.8%), respectively. As the replacement of CCG, the inter-particle interaction will be weakened, which will reduce the thixotropic value and viscosity of the pastes [35].

### 3.2. Setting Time and Compressive Strength

**Figure 5:**
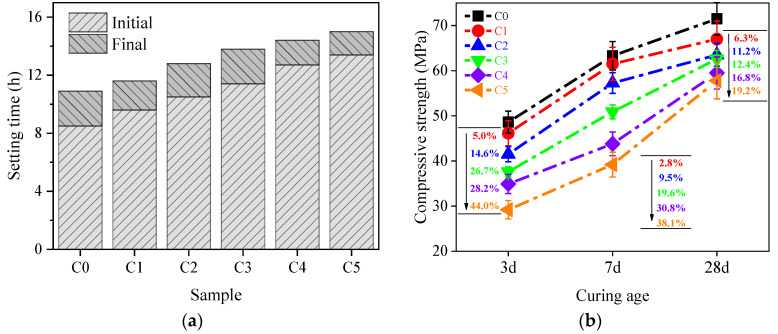
(**a**) Influence of increased amount of CCG on the setting times; (**b**) compressive strength of alkali-activated MK co-blended with CCG.

The setting time of AAM paste with different substitution ratios of CCG is shown in Figure 5a. Clearly, the increased CCG substitution ratio prolonged the setting time of the AAM pastes, which could be caused by the low activity of CCG compared to that of MK.

The compressive strength of samples with different substitution ratios of CCG was tested after curing for 3, 7, and 28 d, and the results are shown in Figure 5b. It shows that along with the increase in the substitution ratio, the compressive strength of hardened AAM paste decreases gradually, especially at 3 and 7 days. Compared to that without CCG (reference, named C0), the strength loss at 3 d and 7 d can reach 44.0% and 38.1%, respectively, for the sample with 50% CCG to replace MK (named C5). Generally, the mechanical properties of AAMs are highly related to the reactivity of the precursor. Despite a higher activity of CCG compared to that of CG after calcination, a large number of inert phases that do not participate in the reaction, such as quartz, are still present, which reduces the compressive strength of the hardened paste [1,36]. However, after curing for 28 d, the strength loss was significantly reduced compared to C0, which is only 19.2%. This indicates that the presence of CCG could not be beneficial for the mechanical property of hardened AAMs at an early age, which is believed to be caused by the low activity of CCG. However, from a long-term aspect, the strength of C5 can be gradually increased, and the compressive strength at 28 d can be nearly 60 MPa, which is enough for a lot of engineering applications.

### 3.3. Calorimetry

**Figure 6:**
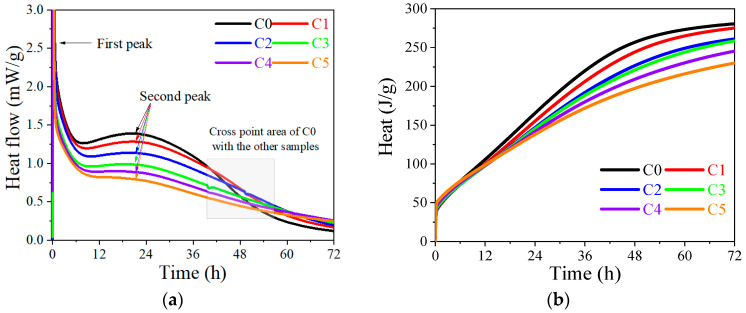
Calorimetric results of alkali-activated MK with the co-blended CCG: (**a**) heat flow; (**b**) cumulative heat.

The heat flow and the cumulative heat for AAMs with different substitution ratios of CCG are shown in Figure 6. Clearly, an obvious exothermic peak can be found in Figure 6a within 1 h, which originates from the wetting and dissolution of the silica–aluminate precursors [37]. The appearance of the second exothermic peak is generally thought to be related to the reaction of precursors under the activation of alkali and the formation of reaction products [38,39].

**Figure 7:**
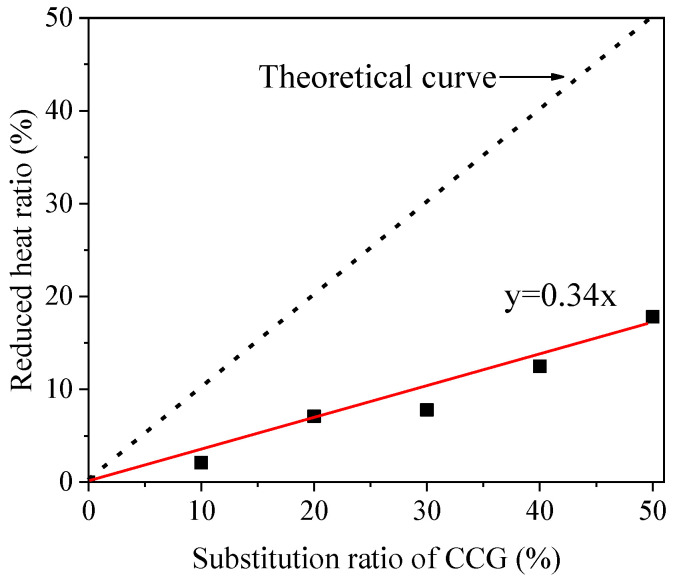
Correlation of the reduced heat ratio with the increased substitution ratio of CCG.

With the increased CCG substitution ratio, the position of the second exothermic peak seems to not be affected, but a gradual decrease in the height of this peak can be found, which indicates that the heat peak is mainly originated from the reaction of MK, and the reduced reaction rate is caused by the decreased amount of MK. After this peak, the heat flow decreased rapidly for C0, but not to the samples with the incorporation of CCG. A cross point can be found at about 40 h for C0 with the other samples, which indicates that the sample with CCG inside has a higher reaction rate than that of C0 after reaction for around 40 h. When the reaction proceeds to 72 h, C5, which has the highest CCG amount, shows the biggest heat flow, followed by C4 to C1, and C0 has the lowest reaction rate. All of these results strongly prove the continuous reaction of CCG and the feasibility of using it as a co-blended material with MK. Figure 6b shows the cumulative heat released after a reaction of 72 h. With the increased amount of CCG, the total released heat gradually decreased from 281 J/g (C0) to 231 J/g (C5), which follows our expectation. Based on Figure 6b, we further calculated the reduced heat ratio and plotted it against the substitution ratio of CCG (Figure 7). The calculation method of the reduced heat ratio is shown below:

(2) $$\mathrm{Reduced}\;\mathrm{heat}\;\mathrm{ratio}=\frac{H_{C0}-H_{\mathrm{Si}}}{H_{C0}}\times 100\%$$

where H_C0_ and H_Si_ refer to the cumulative heat of C0 and samples i (C1–C5).

As can be seen from Figure 7, the reduced heat ratio is significantly lower than the substitution ratio of CCG, which means that there is extra heat provided by the reaction of CCG. If we fit this curve and compare it to the theoretical curve (the dotted line shown in Figure 7, with the assumption that CCG does not react), it indicates that 66% of CCG can react during the first 3 d. This result shows that CCG could also play an important role in contributing to the strength at an early age.

### 3.4. XRD

**Figure 8:**
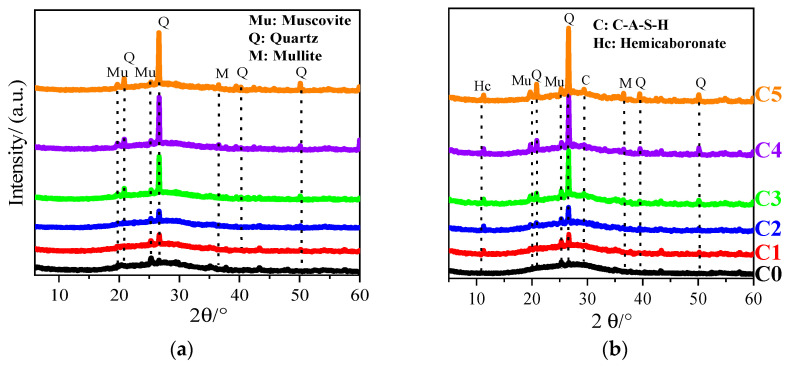
XRD patterns of AAMs with different amounts of CCG after curing for (**a**) 3 d; (**b**) 28 d.

**Figure 9:**
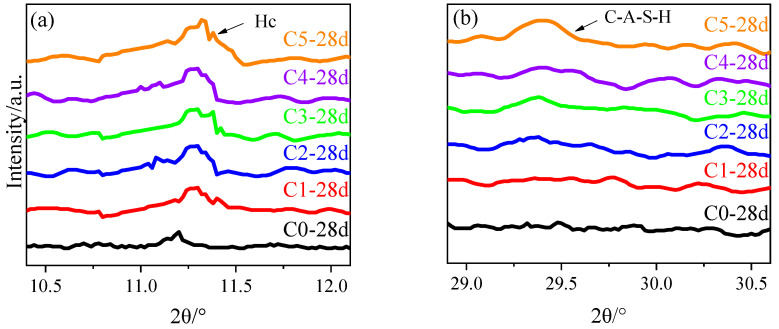
The amplified XRD patterns of alkali-activated MK co-blended with CCG in the range of (**a**) 10.5°–12°; (**b**) 29°–30.5°.

To further understand the effect of CCG on the performance of alkali-activated MK, the XRD patterns of the reaction products in samples after curing for 3 d and 28 d are shown in Figure 8. A broad peak was observed in the 2θ from 20° to 40°, which can be attributed to the amorphous aluminosilicate gels formed after the reaction [40]. Hence, a higher intensity for this broad hump can be found with increased curing age. Additionally, if we compare the effect caused by the addition of CCG, from one aspect, the intensity of the broad hump decreased significantly. In addition, more sharp peaks can be found with the incorporation of CCG. As shown in Figure 1, quartz, muscovite, and mullite are contained in the CCG. Hence, it is not strange to see an increased peak intensity for these minerals inside the hydrated samples. However, some new peaks can be found for the samples with the increased CCG content, namely a peak at around 29.3° and 11.2°, which corresponds to C-A-S-H and hemicarbonate (Hc), respectively [41]. An amplification for these two peaks was made, and it is shown in Figure 9. Clearly, the peak generated from Hc can be found when the CCG content is 10%, but the obvious C-A-S-H peak was only observed when the substitution ratio of CCG is 50%. 

### 3.5. TGA-DTG

**Figure 10:**
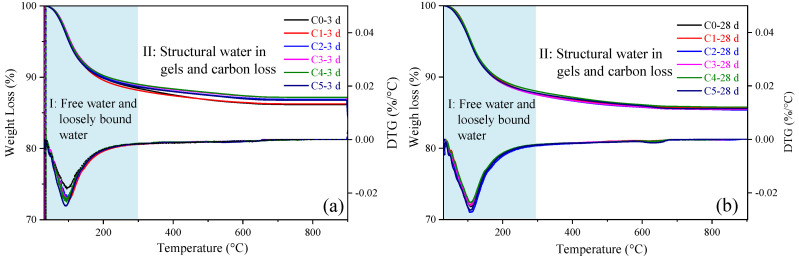
TGA-DTG curves of alkali-activated MK co-blended CCG at the ages of (**a**) 3 d; (**b**) 28 d.

The TGA-DTG curves of different samples at 3 and 28 d are shown in Figure 10. The thermal mass loss of the samples can be explained by two main stages [42,43]. In the first stage (about 25 to 300 °C), the mass loss can be attributed to the evaporation of free water and loosely bound water, which is relatively easily evaporated from gel pores. The second stage of mass loss (from 300 to 890 °C) is caused by all the structural water in the form of -OH in the gels and the carbon in the carbonate. The results of mass loss for each temperature range are shown in Table 4. Firstly, an increased total mass loss of all samples was observed with the increased curing age due to the progressed polymerization. Since there is little difference in the carbonates, the mass loss in the second phase can be mainly related to the structural water in the gel phases [44].

Compared to C0 with the curing age of 3 d, the total mass loss for the other samples at the same curing age is significantly reduced, which indicates that fewer reaction products are formed for these samples with the incorporation of CCG due to its low reactivity. However, with the increased curing age to 28 d, nearly the same mass loss can be found for all of these samples, regardless of the incorporation of CCG or not. It indicates that CCG provides a comparable amount of reaction products with that of C0 after curing for 28 d, which explains the significant increase in strength for the alkali-activated MK co-blended CCG.

### 3.6. Microstructure Analysis

**Figure 11:**
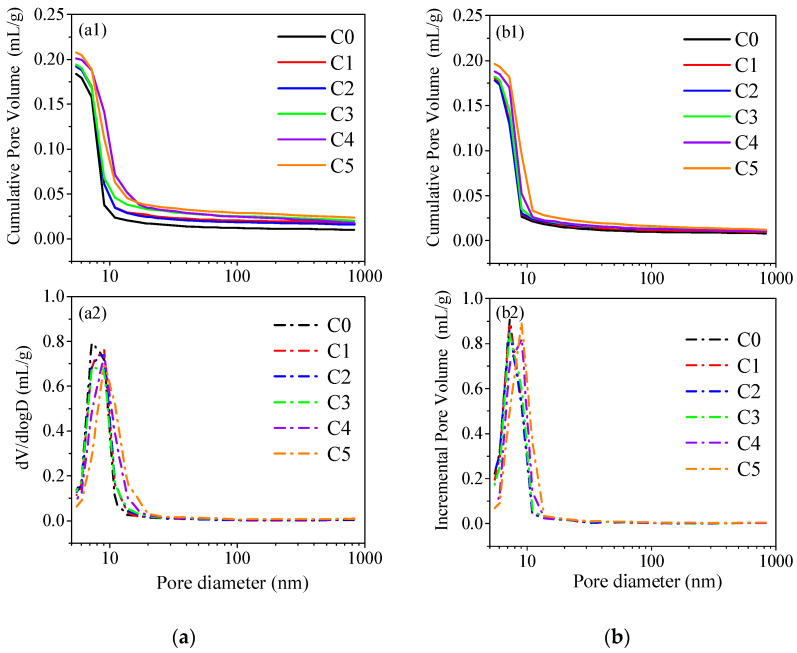
The pore distribution of alkali-activated MK co-blended CCG at the ages of (**a**) 3 d; (**b**) 28 d, wherein 1 refers to the cumulative pore volume and 2 refers to the incremental pore volume.

In order to better understand the mechanical performance of the AAM co-blended with CCG, the microstructure of the hardened samples after curing for 3 d and 28 d is characterized by MIP, and then the pore size distribution and cumulative porosity are shown in Figure 11. Some characterized values for the pore structure are extracted from Figure 11 and shown in Table 5. Generally, depending on the effect of pores on the properties of concrete, the pores in the hardened concrete can be divided into four types, namely the harmless pores (<20 nm), less harmful pores (20–50 nm), harmful pores (50–200 nm), and more harmful pores (>200 nm) [45,46]. As shown in Figure 11, nearly all the pores are under the size of 20 nm, which indicates the formation of a dense structure for the alkali-activated MK.

**Figure 12:**
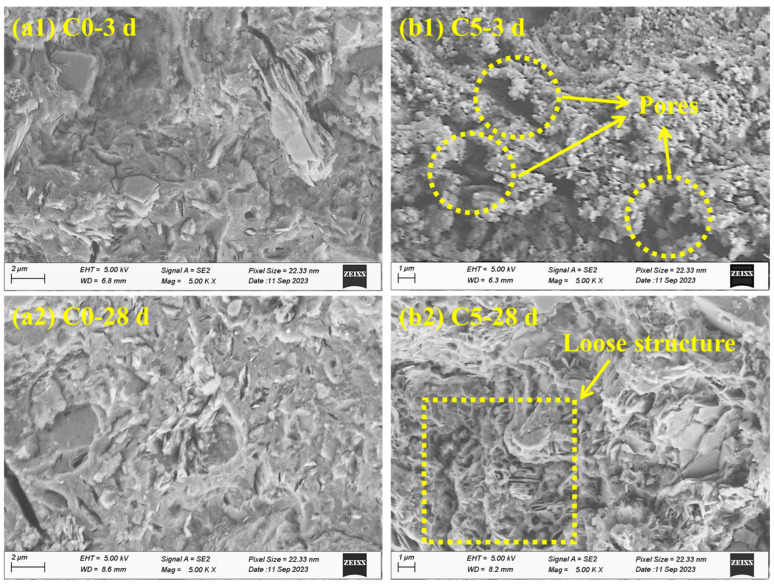
The SEM images of alkali-activated MK co-blended CCG: (**a1**) C0 at 3 d; (**a2**) C0 at 28 d; (**b1**) C5 at 3 d; (**b2**) C5 at 28 d.

Compared to C0, it is obvious to notice an increased trend in the medium pore size and the total porosity through the addition of CCG. This indicates that the incorporation of CCG can lose the microstructure of the hardened paste, which is harmful to the compressive strength of the hardened AAMs. The SEM images also support the above results, in which a loose microstructure was found for C5 no matter whether at 3 d or 28 d, as shown in Figure 12.

However, compared to C5 at 3 d with relatively more pores, C5 at 28 d still shows a dense microstructure. When comparing the difference in porosity for different samples after 3 d and 28 d, it is found that the difference became narrow at 28 d (the difference in porosity at 28 d between C5 and C0 is 1.7%, but at 3 d, this value is 3.2%), which could provide one reason for the narrowed compressive strength of C0 and C5 (shown in Figure 5b). Based on the XRD and TG results, the densified microstructure for the samples with the incorporation of CCG at 28 d could be originated from the formation of reaction products of C-A-S-H or Hc, which can fill in the pores inside the hardened paste and then reduce the porosity.

## 4. Conclusions

In this study, CCG was introduced as a co-blended precursor with MK to prepare AAMs. The following conclusions can be obtained:

Along with the increased replacement amount of MK by CCG, the flowability of the prepared paste increases by up to 28.1% and the viscosity decreases by 55.8% at most, which can reduce the high viscosity and water demand of AAMs prepared with only MK. In addition, a prolonged setting time by up to 31.8% was found with the increased substitution amount of CCG to MK, which can be attributed to the low activity of CCG compared to that of MK. Despite CCG having a certain negative effect on the compressive strength of the hardened AAM paste, especially at ages of 3 and 7 d, this negative effect gradually becomes non-apparent when it comes to late age due to the continuous reaction of CCG. All of these results strongly prove the feasibility of using CCG as one potential material to prepare AAMs.

Besides the performance evaluation, a deep analysis of the reaction process, reaction products, and microstructure was conducted. It was found that AAMs prepared with CCG have a retarded reaction process during the first 3 d. However, it was also found that the incorporation of CCG as the co-blended precursor can produce a certain amount of new reaction products, namely C-A-S-H and hemicarbonate. Although the loose structure is observed in the samples with CCG at an early age, those new reaction products formed at a late age can further decrease the porosity and promote its long-term strength.

## Figures and Tables

**Table 1:** The chemical composition of raw materials (wt.%).

Oxide	SiO_2_	Al_2_O_3_	Fe_2_O_3_	CaO	K_2_O	Na_2_O	MgO	SO_3_	P_2_O_5_	TiO_2_	ZrO_2_	LOI
MK	44.78	50.51	1.51	0.41	0.18	0.08	0.18	0.14	0.09	2.01	0.04	1.02
CCG	56.22	29.11	4.50	3.62	2.45	0.54	1.16	1.08	0.13	0.92	0.02	1.46

**Table 2:** The mean particle diameter of raw materials.

Materials	d50 ^a^ (μm)	D [3,2] ^b^ (μm)	D [4,3] ^c^ (μm)
MK	6.38	3.79	8.57
CCG	13.16	4.12	17.00

^a^ refers to the median diameter; ^b^ refers to the surface mean diameter; ^c^ refers to the volume mean diameter.

**Table 3:** Mixing proportions of different samples.

Samples	Mk Content (wt.%)	CCG Content (wt.%)	Water/Binder	Na_2_O/Binder
C0	100	0	0.6	0.15
C1	90	10	0.6	0.15
C2	80	20	0.6	0.15
C3	70	30	0.6	0.15
C4	60	40	0.6	0.15
C5	50	50	0.6	0.15

**Table 4:** The weight loss of alkali-activated MK co-blended CCG at different temperature ranges.

Samples	Mass Loss (wt. %)					
	3 d			28 d		
	I	II	Sum	I	II	Sum
C0	11.6	2.3	13.9	12.2	2.2	14.4
C1	11.6	1.7	13.7	12.3	2.0	14.4
C2	11.2	1.8	13.0	12.4	2.1	14.6
C3	11.0	1.8	12.8	12.5	2.1	14.5
C4	10.5	1.8	12.3	12.0	2.2	14.2
C5	10.6	1.7	12.3	12.3	2.2	14.4

**Table 5:** Pore structure characteristics of different samples by MIP.

Parameter	Curing Ages	C0	C1	C2	C3	C4	C5
MPD/volume (nm) ^1^	3 d	8.1	8.3	8.3	8.4	9.3	10.0
	28 d	7.8	7.9	7.9	8.0	8.3	9.0
APD/(4 V/A) (nm) ^2^	3 d	8.8	9.5	9.5	9.9	10.7	11.4
	28 d	8.4	8.5	8.6	8.8	9.2	9.9
Porosity (%)	3 d	28.2	28.9	28.9	29.3	30.7	31.4
	28 d	27.4	27.2	27.2	28.0	28.5	29.1

^1^ refers to the medium pore diameter; ^2^ refers to the average pore diameter.

## Data Availability

Data are contained within the article.
